# A Modified Carbon Monoxide Breath Test for Measuring Erythrocyte Lifespan in Small Animals

**DOI:** 10.1155/2016/7173156

**Published:** 2016-05-12

**Authors:** Yong-Jian Ma, Hou-De Zhang, Yong-Qiang Ji, Guo-Liang Zhu, Jia-Liang Huang, Li-Tao Du, Ping Cao, De-Yue Zang, Ji-Hui Du, Rong Li, Lei Wang

**Affiliations:** ^1^Institute of Breath Test Research, Shenzhen University, Shenzhen 518060, China; ^2^Department of Gastroenterology, Nanshan Hospital, Guangdong Medical College, Shenzhen 518052, China; ^3^Institute of Seekya Breath Test Technology, Shenzhen 518053, China; ^4^Shenzhen Testing Center of Medical Devices, Shenzhen 518000, China; ^5^Central Laboratory, Nanshan Hospital, Guangdong Medical College, Shenzhen 518052, China

## Abstract

This study was to develop a CO breath test for RBC lifespan estimation of small animals. The ribavirin induced hemolysis rabbit models were placed individually in a closed rebreath cage and air samples were collected for measurement of CO concentration. RBC lifespan was calculated from accumulated CO, blood volume, and hemoglobin concentration data. RBC lifespan was determined in the same animals with the standard biotin-labeling method. RBC lifespan data obtained by the CO breath test method for control (CON, 49.0 ± 5.9 d) rabbits, rabbits given 10 mg/kg·d^−1^ of ribavirin (RIB10, 31.0 ± 4.0 d), and rabbits given 20 mg/kg·d^−1^ of ribavirin (RIB20, 25.0 ± 2.9 d) were statistically similar (all *p* > 0.05) to and linearly correlated (*r* = 0.96, *p* < 0.01) with the RBC lifespan data obtained for the same rabbits by the standard biotin-labeling method (CON, 51.0 ± 2.7 d; RIB10, 33.0 ± 1.3 d; and RIB20, 27.0 ± 0.8 d). The CO breath test method takes less than 3 h to complete, whereas the standard method requires at least several weeks. In conclusion, the CO breath test method provides a simple and rapid means of estimating RBC lifespan and is feasible for use with small animal models.

## 1. Introduction

Some kinds of anemia and jaundice in variety of diseases are attributed to hemolysis, a condition characterized by excessively rapid destruction of erythrocytes (RBCs) in circulating blood circulation or bone marrow. The gold standard for diagnosis of hemolysis is a reduced RBC lifespan [1]. Recently, a noninvasive carbon monoxide (CO) breath test for indexing of human RBC lifespan has become available. The method is simple and rapid, enabling hemolysis to be detected in clinical practices more easily than ever before [2–8].

Animal models are critical for elucidating pathological mechanisms and developing effective therapies. Animal studies of hemolysis, however, have relied mainly on indirect measures, such as changes in peripheral blood reticulocyte percentage or serum bilirubin concentration. Accurate hemolysis detection in animals has been limited by the fact that the standard techniques for measuring RBC lifespan, such as ^51^Cr and biotin-labeling tests, are complex and time-consuming [1]. Furthermore, the protocol for human CO breath testing has not been translated into a protocol appropriate for animal subjects.

The aim of the present study was to develop a CO breath test protocol suitable for measuring RBC lifespan in rabbits. The long-term goal of this work is to provide a convenient means for assessing hemolysis in small animals.

## 2. Materials and Methods

### 2.1. Animal Model

Chronic hemolytic rabbit models were prepared by extended ribavirin administration. Long-term ribavirin treatment, as is used for the treatment of hepatitis C, produces the major side effect of hemolytic anemia [9–11]. Adult male and female New Zealand white rabbits (2.2–2.7 kg) were purchased from Guangdong Medical Laboratory Animal Center and housed in standard animal labs of Shenzhen Medical Devices Testing Center. The research protocol was approved by the Laboratory Animal Ethics Committee of Shenzhen Institute for Drug Control. Fifteen rabbits were divided randomly into a control (CON) group, a low-dose ribavirin treatment (RIB10) group, and a high-dose ribavirin treatment (RIB20) group (*N* = 5 per group). The RIB10 and RIB20 treatment group rabbits were given ribavirin (dissolved in 5 mL of 5% glucose solution) 10.0 and 20.0 mg/kg per day by intragastric administration. The CON group rabbits were given only the vehicle solution (5 mL of 5% glucose solution) per day by intragastric administration. The rabbits were subjected to blood tests and RBC lifespan measurement with the CO breath test on experimental day 20. RBC lifespan measurement by the biotin-labeling method commenced on experimental day 21. The rabbits were given ribavirin (RIB10 and RIB20 groups) or vehicle solution (CON group) during the whole biotin-labeling measurement period. The timeline employed for our RBC lifespan measurements was based on prior observations in our laboratory of declining hemoglobin concentrations, stabilized by 3 weeks of ribavirin treatment (data not shown).

Blood samples were collected from the marginal ear vessels for blood tests (i.e., hemoglobin concentration, hematocrit, reticulocyte count, and serum concentration of total bilirubin and indirect bilirubin by standard methods) one day before commencing with administration of ribavirin (or vehicle) and on day 20 when the RBC lifespan measurement by the CO breath test was carried out. The collected blood samples were also used for blood volume estimation as described below.

### 2.2. Evans Blue Dye (EBD) Dilution Test

The EBD dilution test was used to estimate blood volume [12, 13]. It was conducted on the same day of the CO breath test after breath sampling. Blood volume is a parameter in the equation used to calculate RBC lifespan based on CO breath test results. The EBD dilution test was performed as described elsewhere with slight modifications [12, 13]. Briefly, for each animal, an initial (blank) 1 mL blood sample was withdrawn from the auricular artery and 1 mL of 0.2% EBD solution in normal saline was injected into the marginal vein. Subsequently, 0.5 mL of saline was used to wash out the injected dye. After 8 min, another 1 mL blood sample was withdrawn. All samples were collected in heparinized tubes and plasma was separated from whole blood. EBD content was determined by a spectrophotometric assay, wherein a volume of 0.2 mL plasma of each blood sample was placed in a 96-well microplate and the absorbance was detected at 650 nm with microplate reader (SpectraMax M3, Molecular Devices, USA). Plasma volume was calculated based on the dilution principle (plasma volume = volume of injected EBD × concentration of injected EBD/concentration of EBD of measured sample). Finally, estimation of blood volume was calculated from the plasma volume determined by the preceding formula and hematocrit level (blood volume = plasma volume/1 − hematocrit).

### 2.3. CO Breath Test

**Figure 1:**
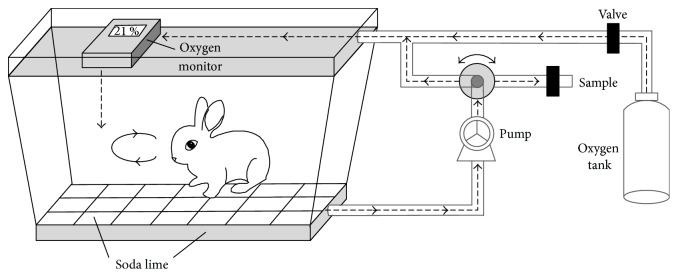
Schematic representation of the rebreath system for accumulating endogenous CO of rabbits.

On day 20 of the ribavirin (or vehicle) treatment, each animal's CO breath test was conducted in our rebreath apparatus (constructed in our laboratory). As shown in Figure 1, the rebreath apparatus consists of a 90-L sealed biocage and ventilation accessories. The experimental procedure involved the six steps outlined below:

(1) Soda lime (500 g), a CO_2_ absorbent material, was placed into the bottom of the cage (replaced every 7 h) and the cage was sealed closed.

(2) A 1.5-L baseline air sample was collected from the cage by pumping the cage air into an aluminum bag through a gas circuit connector.

(3) The experimental subject was placed into the cage after being weighed and the cage was closed after the oxygen supply and the air circulation pump were switched on. Air flowed through the cage from top to bottom at rate of 8.0 L/min. The O_2_ tension level was monitored and maintained at 20~23% by adjusting the oxygen supply flow (~4 mL/min). In this closed system, the experimental rabbit repeatedly exhaled and inhaled the same gas. While the consumption of O_2_ was supplemented, exhaled CO_2_ was absorbed, and endogenous CO accumulated in the cage.

(4) Another 1.5-L gas sample was collected from the cage following 120 min of rebreath accumulation.

(5) CO concentrations (ppm) in the baseline control and experimental gas were analyzed by CO infrared spectroscopy (RBC lifespan analyzer RBCS-01, Seekya Biotec. Shenzhen, China). The difference between the baseline control and experimental samples was taken as the accumulated concentration of endogenous CO (P_CO_).

(6) RBC lifespan (in days) was calculated based on CO measurements from the following formula, which equates mean RBC lifespan with the total capacity of CO from hemoglobin divided by the CO quantity released per day:

(1) $$\begin{array}{c} \text{RBC life span}=\frac{\left(\left(V_{b}\times \text{Hb}\times 4\times 22.4\right)/64400\right)}{\left(\left(V_{\text{cage}}\times {Ρ}_{\text{CO}}\times 10^{-6}\times 0.7\times 24\right)/t\right)}, \end{array}$$

where *V*_*b*_ is blood volume in liters and Hb is hemoglobin concentration in g/L. *V*_*b*_ and Hb are multiplied by molar ratio of CO to hemoglobin (i.e., 4) and the number of liters consumed by 1 mole of CO (i.e., 22.4); the product is divided by the molecular weight of hemoglobin (i.e., 64,400) to give the total capacity of CO from hemoglobin. The cage volume in liters, *V*_cage_, is multiplied by *Ρ*_CO_ as well as by a conversion factor from ppm to L (i.e., 10^−6^). The hemoglobin turnover in rabbit was set to 0.7, based on approximation from humans [2, 14]. The mean endogenous CO production fraction in rabbit derived by the standard biotin-label method was 0.68 (range, 0.65–0.75) and 24 is hours of a day; the product is divided by the rebreath time in hours (*t*) to give the CO quantity released per day. Because the *V*_cage_ is 90 L and *t* is 2.0 h in our experiment, the formula was simplified to the following expression:

(2) $$\begin{array}{c} \text{RBC life span}=\frac{V_{b}\times \text{Hb}\times 1.84}{{Ρ}_{\text{CO}}} \end{array}$$

in which the variables are defined as in (1).

### 2.4. Biotin Labeling and Flow Cytometry

Reinfusion of autologous biotin-labeled RBCs and flow cytometric quantitation was used as a reference standard for RBC lifespan quantitation. The test began on day 21, the day after CO breath testing. The procedures were carried out as described elsewhere with some modifications [15, 16]. Briefly, about 3.5 mL of blood obtained from an ear marginal vein of each rabbit was collected in a heparinized, sterilized tube. Under aseptic conditions, the blood was washed three times with 7 mL Dulbecco's PBS (D-PBS; #14190-045; Gibco) and then resuspended in 5.0 mL of D-PBS. Finally, 3.1 mL of blood was transferred to a 50 mL tube. A 40 *μ*L aliquot of stock solution containing 2 *μ*g/*μ*L N-hydroxysuccinimide biotin (H1759FD; Sigma, USA) in 10% DMSO (Sigma, USA) was added to the suspension and the tube was incubated at room temperature for 30 min to allow biotin conjugation. The biotin-labeled RBCs were washed three more times with 8-fold volumes of D-PBS each time. The washed blood cells were resuspended in 3.1 mL of D-PBS. After the ratio of biotin-labeled RBCs was confirmed to be >98% by flow cytometry, the suspension was infused completely into the ear marginal vein. The amount of time interval from the initial blood collection to reinfusion was no more than 6 h. Flow cytometry was performed exactly as prescribed in the manufacturer's protocol (Cytomics FC 500, Beckman Coulter, USA).

The biotin-labeled RBC ratio in blood samples collected 1 h after reinfusion was determined as the initial ratio. Additional blood samples were taken 1 d, 1 wk, and 2 wks after reinfusion. Subsequently, samples were taken weekly for the CON group and every 3 d for the RIB10 and RIB20 groups until the biotin-labeled RBC ratio was <20%. The percentage of biotinylated RBCs remaining in circulation was plotted against the time that had elapsed since transfusion. Biotin-labeled RBC ratio was plotted as a function of time in a scatterplot. Survival curves were fit linearly with least-squares regression curves. The mean potential RBC lifespan was calculated as the intersection of this linear regression of all data points and the time axis [16].

### 2.5. Statistical Analysis

Statistical analyses were performed in Excel version 2013 (Microsoft, USA). Data are represented as means ± SDs. Measurements of P_CO_ were compared with paired *t*-tests. A correlation coefficient was calculated with the Spearman correlation test, and the coefficient of variation was calculated by dividing the SD by the mean.

## 3. Results

Physiological data obtained for each group are reported in Table 1. Briefly, there were no group differences in body weight or blood volume. Regarding hemolytic parameters, a modest but significant reduction in hemoglobin concentration was observed in the RIB20 treatment group, a robust augmentation of reticulocytes was observed in both treatment groups in a dose-dependent manner, and both total bilirubin and indirect bilirubin quantities were markedly increased in both treatment groups. The accumulated concentration of exhaled CO showed an increasing trend with increasing ribavirin treatment dosage, though the group differences did not reach statistical significance.

**Figure 2:**
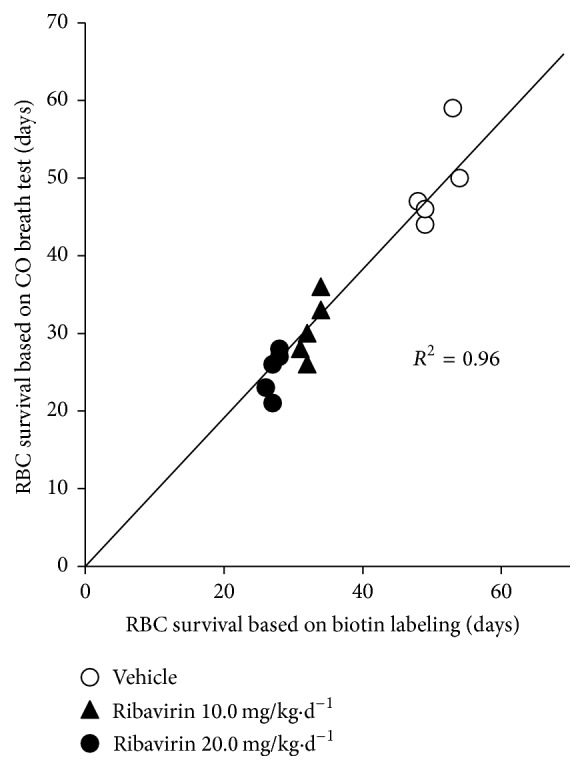
Comparison of the red blood cell (RBC) life span calculated by the CO breath test (*Y*) and the biotin-labeling measurement (*X*) in 15 rabbits with different statuses. The two methods show a very strong direct correlation (*R* ^2^ = 0.96, *p* < 0.001).

The RBC lifespan results are reported in Table 2. Briefly, ribavirin treatment produced a dose-dependent reduction in RBC lifespan. The lifespan datum obtained from the CO breath test method did not differ significantly from that obtained from the standard biotin-labeling method for any of the three groups. Furthermore, as shown in Figure 2, the CO breath test data exhibited a highly significant positive linear correlation with the biotin-labeling data, with all data points approximating a least-squares regression line with a slope of 1.0, indicating that the RBC lifespan values calculated by the two methods were almost equal. RBC lifespan measurement by the CO breath test took no more than 4 hours in total, whereas the mean time periods for the biotin-labeling tests were 45 days, 28 days, and 20 days for the CON, RIB10, and RIB20 groups, respectively.

## 4. Discussion

In this study, RBC lifespan calculated based on accumulated CO concentration measured with our newly developed rebreath system, blood volume data, and hemoglobin concentration data matched standard biotin-labeling measurement data in hemolytic anemic rabbit models prepared by chronic ribavirin administration. These results demonstrate that RBC lifespan data calculated from the CO breath test can be used in animal studies, reducing the period of time needed to obtain RBC lifespan data from several weeks, with the standard biotin-labeling method, to just a few hours.

The classic standard methods for the determination of RBC lifespan are reinfusion measurements of the labeled RBC including ^51^Cr-labeling developed in the 1950s and biotin-labeling developed in the 1980s, which involve* ex vivo* labeling and reinfusion of autologous RBCs followed by measures of the length of time during which the labeled RBCs remain in circulation [1]. RBC labeling can be done with ^51^Cr, which binds tightly but noncovalently to hemoglobin; ^51^Cr-labeled RBCs are then detected by radioactive quantitation. Alternatively, RBC membranes can be labeled nonisotopically with biotin, as was done in this study; biotin-labeled RBCs are then detected by secondary labeling with fluorochrome-conjugated streptavidin and flow cytometry. Both of these labeling methods have the disadvantage of requiring multiple venesections over a period of several weeks or even several months in some cases. As a result, neither is suitable for animal and clinical studies. The reason underlying elevated RBC turnover in anemic patients is, in most cases, not known with confidence.

CO is an* in vivo* catabolic byproduct of heme that originates from the stoichiometric conversion of the *α*-methene carbon of the porphyrin ring to CO during the catabolism of heme to bilirubin. Because 70~80% of heme turnover is due to hemoglobin breakdown, CO production reflects RBC turnover [17]. A half century ago, using a complicated rebreath system and continuous monitoring of blood carboxyhemoglobin (COHb) concentrations, Coburn et al. [18] demonstrated that the quotient of the total CO capacity of all hemoglobin degradation divided by the amount of CO produced daily provides an accurate index of the mean RBC lifespan. Because the lungs are the only excretory route for CO, exhaled CO provides a measure of RBC turnover if environmental CO contamination is eliminated. In 1992, Strocchi et al. [2, 3] described a simple noninvasive CO breath test for estimating RBC lifespan in humans. In Strocchi's test, an alveolar breath sample is obtained by a single deep exhalation after 20 s of breath holding. The endogenous component of alveolar CO is then determined by subtracting the P_CO_ of the environmental air from that of the alveolar breath sample; and, then, RBC lifespan is calculated from the endogenous *Ρ*_CO_ and hemoglobin concentration data. Several clinical observations have demonstrated that Strocchi's test has good consistency with RBC survival status [2–8]. Furthermore, breath CO monitoring has been proposed as a golden standard for neonatal hemolytic jaundice [19].

Strocchi's protocol is not practical for small animals because it would be very difficult to collect an alveolar breath sample from them. To overcome this challenge, we developed a simple apparatus for CO collection from rabbits based on a rebreath mechanism (Figure 1). Instead of monitoring blood COHb concentration continuously to determine the production rate of endogenous CO as described by Coburn et al. [18], our method measures expired endogenous CO that has been allowed to accumulate in a sealed box. We have found that accumulated expired CO differs significantly from baseline CO levels within 2 hours of rebreathing (data not shown). Theoretically, our CO analysis principle is also suitable for rats and mice. However, the apparatus may need to be redesigned to better accommodate them. In particular, an alternative CO analysis method, such as gas chromatography, may be more appropriate for their small breath volumes. Compared to the calculation formula in Strocchi's protocol, the protocol for our method, like the Coburn protocol, requires the parameter of blood volume in addition to the CO and hemoglobin parameters. However, the EBD dilution technique for measuring a rabbit's blood volume is quite simple and can be completed rapidly with automatic instruments. As shown in Figure 2, RBC lifespan data calculated based on our modified CO breath test was virtually identical to the data obtained by the standard biotin-labeling method. These results demonstrate that our modified CO breath test for estimation of RBC lifespan, which combines the advantages of Coburn's and Strocchi's protocols, is reliable and feasible for use in animal studies.

An effective method for rapid measurement of RBC lifespan in small animals would be helpful in a variety of RBC study settings, including RBC aging studies and studies examining hemolytic mechanisms, as well as antihemolytic drug development. In fact, researchers at our institution are proceeding with antihemolysis drug screening employing our CO breath test in hemolytic rabbit models.

## 5. Conclusion

RBC lifespan data calculated based on accumulated CO concentration measured with the rebreath system, blood volume determined by the EBD dilution technique, and hemoglobin concentration are virtually identical to RBC lifespan data obtained by the standard biotin-labeling method. The rebreath system enables RBC lifespan to be determined within a few hours rather than in several weeks as is needed with standard RBC labeling approaches. The present findings demonstrate that our method of RBC lifespan calculation based on CO breath test data is feasible for use in animal studies.

## Figures and Tables

**Table 1:** Physiological parameters of the groups on day 20 of ribavirin treatment.

Group	*N*	Weight (kg)	Blood volume (mL)	Hemoglobin (g/L)	Reticulocyte (%)	Bilirubin (*μ*mol/L)		CO (ppm)
						Total	Indirect	
CON	5	2.7 ± 0.2	186 ± 54	117 ± 1.1	2.1 ± 0.4	0.8 ± 0.09	0.3 ± 0.11	0.81 ± 0.19
RIB10	5	2.7 ± 0.3	130 ± 17	117 ± 5.7	6.5 ± 0.8^*∗∗*^	1.5 ± 0.28^*∗∗*^	0.8 ± 0.13^*∗∗*^	0.92 ± 0.18
RIB20	5	2.8 ± 0.2	128 ± 14	107 ± 6.6^*∗*#^	11.7 ± 1.8^*∗∗*,##^	1.7 ± 0.11^*∗∗*^	1.1 ± 0.09^*∗∗*^	1.00 ± 0.05

^*∗*^ *p* < 0.05, ^*∗∗*^ *p* < 0.01 versus CON. ^#^ *p* < 0.05, ^##^ *p* < 0.01 versus RIB10.

**Table 2:** Comparison of RBC lifespan determined by biotin labeling versus CO breath test methods.

Group	*N*	RBC lifespan (d)		*p*
		Biotin labeling	CO breath test	
CON	5	51 ± 2.7	49 ± 5.9	0.515
RIB10	5	33 ± 1.3^*∗∗*^	31 ± 4.0^*∗∗*^	0.200
RIB20	5	27 ± 0.8^*∗∗*##^	25 ± 2.9^*∗∗*^	0.108

^*∗∗*^ *p* < 0.01 versus CON. ^##^ *p* < 0.01 versus RIB10.

## References

[1] Franco R. S. (2012). Measurement of red cell lifespan and aging. *Transfusion Medicine and Hemotherapy*.

[2] Strocchi A., Schwartz S., Ellefson M., Engel R. R., Medina A., Levitt M. D. (1992). A simple carbon monoxide breath test to estimate erythrocyte turnover. *The Journal of Laboratory and Clinical Medicine*.

[3] Furne J. K., Springfield J. R., Ho S. B., Levitt M. D. (2003). Simplification of the end-alveolar carbon monoxide technique to assess erythrocyte survival. *Journal of Laboratory and Clinical Medicine*.

[4] Virtue M. A., Furne J. K., Nuttall F. Q., Levitt M. D. (2004). Relationship between GHb concentration and erythrocyte survival determined from breath carbon monoxide concentration. *Diabetes Care*.

[5] Virtue M. A., Furne J. K., Ho S. B., Levitt M. D. (2004). Use of alveolar carbon monoxide to measure the effect of ribavirin on red blood cell survival. *American Journal of Hematology*.

[6] Mitlyng B. L., Chandrashekhar Y., Furne J. K., Levitt M. D. (2006). Use of breath carbon monoxide to measure the influence of prosthetic heart valves on erythrocyte survival. *The American Journal of Cardiology*.

[7] Mitlyng B. L., Singh J. A., Furne J. K., Ruddy J., Levitt M. D. (2006). Use of breath carbon monoxide measurements to assess erythrocyte survival in subjets with chronic diseases. *American Journal of Hematology*.

[8] Medina A., Ellis C., Levitt M. D. (1994). Use of alveolar carbon monoxide measurements to assess red blood cell survival in hemodialysis patients. *American Journal of Hematology*.

[9] Hézode C., Bronowicki J. (2016). Ideal oral combinations to eradicate HCV: the role of ribavirin. *Journal of Hepatology*.

[10] Tod M., Farcy-Afif M., Stocco J. (2005). Pharmacokinetic/pharmacodynamic and time-to-event models of ribavirin-induced anaemia in chronic hepatitis C. *Clinical Pharmacokinetics*.

[11] Brochot E., François C., Castelain S. (2012). A new tool to study ribavirin-induced haemolysis. *Antiviral Therapy*.

[12] Armin J., Grant R. T., Pels H., Reeve E. B. (1952). The plasma, cell and blood volumes of albino rabbits as estimated by the dye (T 1824) and 32P marked cell methods. *The Journal of Physiology*.

[13] Baby P. M., Kumar P., Kumar R. (2014). A novel method for blood volume estimation using trivalent chromium in rabbit models. *Indian Journal of Plastic Surgery*.

[14] Vreman H. J., Wong R. J., Stevenson D. K., Penney D. G. (2000). Carbon monoxide in breath, blood, and other tissues. *Carbon Monoxide Toxicity*.

[15] Russo V., Barker-Gear R., Gates R., Franco R. (1992). Studies with biotinylated RBC: (1) use of flow cytometry to determine posttransfusion survival and (2) isolation using streptavidin conjugated magnetic beads. *Advances in Experimental Medicine and Biology*.

[16] Mock D. M., Matthews N. I., Zhu S. (2011). Red blood cell (RBC) survival determined in humans using RBCs labeled at multiple biotin densities. *Transfusion*.

[17] Coburn R. F. (2012). The measurement of endogenous carbon monoxide production. *Journal of Applied Physiology*.

[18] Coburn R. F., Williams W. J., Kahn S. B. (1966). Endogenous carbon monoxide production in patients with hemolytic anemia. *Journal of Clinical Investigation*.

[19] American Academy of Pediatrics Subcommittee on Hyperbilirubinemia (2004). Management of hyperbilirubinemia in the newborn infant 35 or more weeks of gestation. *Pediatrics*.

